# The distribution of N-acetyltransferase 2 genotypes in a cohort of tuberculosis patients in a single center in Henan Province of China

**DOI:** 10.1186/s12879-026-14424-x

**Published:** 2026-09-22

**Authors:** Jiankang Li, Liangyu Chen, Hongmei Shi, Chang Zhao, Peicong Xu, Yanyan Mo, Xin Liu, Yihan Zhao, Ruixia Liang

**Affiliations:** 1 Department of Tuberculosis/Criticalllness Area, Henan Infectious Diseases (TB) Clinical Research Center, Henan Provincial Chest Hospital, No.1, Weiwu·Road, Zhengzhou, Henan 450001 China; 2 College of Public Health, Zhengzhou University, Zhengzhou, 450001 China

**Keywords:** Tuberculosis, Isoniazid, N-acetyltransferase 2, Pharmacogenomics, Acetylator phenotype, Liver function abnormality

## Abstract

**Background:**

Genetic variation in N-acetyltransferase 2 (NAT2) affects isoniazid metabolism, but acetylator distributions and their association with liver abnormalities remain incompletely characterized in central China. We described genotype-inferred NAT2 acetylator status and assessed its association with new in-hospital liver function abnormality among patients with tuberculosis.

**Methods:**

This retrospective, single-center study included 801 patients who underwent NAT2 genotyping at Henan Provincial Chest Hospital between January 2021 and May 2024. The overall cohort was used to describe slow (SA), intermediate (IA), and fast (FA) acetylator distributions. The primary analysis included 554 patients with normal liver function at admission and a recorded isoniazid dose; 690 patients with normal baseline liver function formed the secondary cohort. The primary comparison was SA versus non-SA. Logistic regression adjusted for age, sex, and recorded isoniazid dose, with additional and sensitivity analyses.

**Results:**

Among 801 patients, 369 (46.1%) were IA, 278 (34.7%) were FA, and 154 (19.2%) were SA. In the primary analysis, 143 patients developed a new in-hospital liver function abnormality. The event rate was 28.7% (29/101) in SA and 25.2% (114/453) in non-SA (crude odds ratio [OR], 1.20; 95% confidence interval [CI], 0.73–1.92; *P* = 0.462). After adjustment for age, sex, and recorded isoniazid dose, SA was not significantly associated with the outcome (adjusted OR, 1.18; 95% CI, 0.72–1.89; *P* = 0.507). Additional adjustment for pulmonary tuberculosis and isoniazid susceptibility produced the same estimate (OR, 1.18; 95% CI, 0.72–1.90; *P* = 0.505). Secondary and sensitivity analyses were consistent. At 80% power, the approximate minimum detectable OR for a risk-increasing association was 1.94.

**Conclusions:**

Slow acetylator status was not significantly associated with new in-hospital liver function abnormality, although smaller or moderate effects could not be excluded. Because the outcome was not causally adjudicated ATB-DILI and systematic pharmacokinetic and longitudinal treatment data were unavailable, these findings do not establish the clinical utility of NAT2-guided dosing. Prospective multicenter validation is needed.

**Supplementary Information:**

The online version contains supplementary material available at https://doi.org/10.1186/s12879-026-14424-x.

## Introduction

Tuberculosis (TB), a chronic infectious disease caused by *Mycobacterium tuberculosis*, remains a major global public health concern [1]. The global use of standardized first-line anti-tuberculosis treatment regimens is measured in hundreds of millions [2]. However, annual reports from the World Health Organization (WHO) indicate that the success rate for drug-susceptible tuberculosis treatment is approximately 90%. About 10% of patients with drug-susceptible tuberculosis experience treatment failure, suggesting that bacterial susceptibility is not the sole factor determining treatment outcomes [1]. Moreover, the management of tuberculosis is increasingly complicated by multiple factors, ranging from acquired resistance to novel agents like bedaquiline to complex host-pathogen immunological interactions [3–5]. Therefore, optimizing the efficacy of foundational first-line drugs like isoniazid through a personalized medicine approach remains clinically important. For a long time, the contribution of patient heterogeneity to tuberculosis treatment outcomes has not been fully characterized, mainly due to the absence of clinical technologies capable of identifying key sources of heterogeneity [6, 7].

Isoniazid (INH) is a cornerstone of the first-line anti-tuberculosis regimen, with strong early bactericidal activity among available anti-TB drugs, as well as low cost and broad accessibility. The INH acetylator status of patients is an important source of host-related heterogeneity that may influence anti-TB treatment efficacy [8]. The main metabolic pathway of INH in the human body is through catalysis by N-acetyltransferase 2 (NAT2), which acetylates it into the inactive acetylisoniazid [9, 10].The 870 bp coding region of the NAT2 gene harbors numerous variants, with seven common single-nucleotide polymorphisms (SNPs) being particularly relevant to phenotype determination. Key SNPs such as rs1801279 (191G > A), rs1041983 (282 C > T), rs1801280 (341T > C), and rs1799930 (590G > A) are established core loci for NAT2 genotyping due to their high prevalence across populations [11]. Specific alleles, notably rs1799930 (590G > A) and rs1799931 (857G > A), are strongly associated with the slow acetylation phenotype, as they lead to a significant decrease in enzyme activity [12]. Research confirms that these SNPs and their derived haplotypes (e.g., NAT25, NAT26, NAT27) are primary determinants of interindividual differences in NAT2 enzyme metabolic capacity [13].

Single nucleotide polymorphisms (SNPs) in the NAT2 gene lead to significant differences in enzyme activities. In general, an individual’s acetylation phenotype is determined by the combination of two NAT2 alleles. Based on the SNPs, the population can be classified into three genotypes: fast acetylator (FA), intermediate acetylator (IA), and slow acetylator (SA) [14, 15]. Slow Acetylator (SA) typically refers to individuals carrying two slow-acetylation alleles; for instance, patients with NAT2*5/*5, NAT2*5/*6, NAT2*5/*7, NAT2*6/*6, NAT2*6/*7, or NAT2*7/*7 allele combinations are likely to exhibit the slow acetylator phenotype [13]. Owing to significantly reduced NAT2 enzyme activity, slow acetylators show decreased clearance of isoniazid, resulting in elevated drug exposure and an increased risk of peripheral neuropathy and hepatotoxicity [2, 6–8]. Multiple meta-analyses have indicated that the risk of anti-tuberculosis drug-induced liver injury (ATDILI) is significantly higher in NAT2 slow acetylators than in fast and intermediate acetylators [9, 10, 14, 15]. IA typically refers to individuals carrying one fast acetylation allele and one slow acetylation allele, such as the combinations of NAT2*4/*5, NAT2*4/*6, or NAT2*4/*7, and the enzyme activity of intermediate acetylators is between that of slow acetylators and fast acetylators [13]. FA typically refers to individuals carrying two fast acetylation alleles, such as NAT2*4/*4 [13]. FA have high levels of NAT2 enzyme activity and can rapidly metabolize isoniazid. However, fast acetylators may face the risk of treatment failure because excessively fast clearance of isoniazid may lead to insufficient drug exposure [16]. For example, a study conducted in northern Thailand found that the NAT2 fast acetylator phenotype was associated with an increased risk of tuberculosis retreatment [17, 18].

Several studies have explored NAT2 genotyping as a potential tool for optimizing isoniazid dosing; however, routine implementation remains limited by insufficient prospective evidence [15, 19]. Nevertheless, due to the previous lack of feasible and convenient methods to determine INH acetylator status, this strategy was rarely deployed in clinical practice. Therefore, to bridge this translational gap and considering the distinct genetic profiles across populations, local epidemiological data are urgently required. Henan is a populous province in China and has a heavy burden of tuberculosis. However, there is currently a lack of research data on NAT2 genotyping. In this study, we describe the distribution of INH acetylator phenotypes in a single-center cohort of tuberculosis patients from a single provincial chest hospital in Henan, with the aim of providing evidence for the potential development of host-based INH dose-adjustment strategies.

## Methods

### Study design and analysis populations

This retrospective, single-center study identified patients with tuberculosis who underwent NAT2 genotyping at Henan Provincial Chest Hospital between January 2021 and May 2024. After eligibility screening, 801 patients constituted the overall cohort used to describe baseline characteristics and the distribution of genotype-inferred NAT2 acetylator status. For the revised liver-function analyses, the baseline-normal cohort comprised 690 participants with normal liver function at admission and was used for secondary and exploratory analyses. The primary analysis set comprised 554 participants with normal liver function at admission and a recorded INH dose. The primary comparison was SA versus non-SA (IA and FA combined); separate IA-versus-SA and FA-versus-SA comparisons were secondary analyses. The study protocol was approved by the Ethics Committee of Henan Provincial Chest Hospital, which waived the requirement for informed consent because of the retrospective design.

#### Inclusion criteria

(1) Patients with a documented diagnosis of tuberculosis who were admitted to Henan Provincial Chest Hospital between January 2021 and May 2024; (2) Completion of NAT2 genotyping during hospitalization, with a valid and interpretable genotyping result; and (3) availability of the key demographic and clinical information required for the overall descriptive analyses.

#### Exclusion criteria

(1) Severe comorbidities that could substantially affect clinical assessment, including severe heart failure, severe malnutrition, or human immunodeficiency virus infection; (2) Long-term use of concomitant non-antituberculosis medications with a known risk of liver injury before or during antituberculosis treatment; and (3) Missing key demographic or diagnostic information required for the overall descriptive analyses; isoniazid allergy; or unstable vital signs, such as shock or severe respiratory failure, requiring immediate emergency management.

### NAT2 genotyping test

The genomic DNA of the peripheral blood samples was extracted using the BloodGen Mini Kit (CW biotech, Beijing, China). After the DNA preparation, the NAT2 genotype was determined using a high-resolution melting amplification kit (Zeesan Biotech Co., Xiamen, China) [20]. Four SNPs HRM assays which focus on the SNPs analysis of rs1801280 (341T > C), rs1799929 (481 C > T), rs1799930 (590G > A) and rs1799931 (857G > A) in an 870-bp coding region of NAT2 were conducted. The PCR and HRM were performed according to the method recommended by the manufacturer [20] on SLAN^®^-96 S (HONGSHI, China). The HRM curve was analyzed using the SLAN^®^-96 S Realtime PCR System release SLAN 8.2.2.

Briefly, distinct mutational profiles at the four detected SNP loci were matched to canonical NAT2 haplotypes and allelic variants. The wild-type allele NAT2*4 corresponds to the absence of the above four mutations and confers rapid acetylation capacity, whereas the presence of characteristic variant combinations defines the slow-acetylation alleles including NAT2*5, NAT2*6, and NAT2*7. On the basis of NAT2 diploid genotypes, individuals were classified into three acetylation phenotypes: fast acetylators (FA) carried two wild-type rapid alleles (NAT2*4/*4); intermediate acetylators (IA) harbored one rapid allele and one slow allele (e.g., NAT2*4/*5, NAT2*4/*6, NAT2*4/*7); slow acetylators (SA) possessed two slow variant alleles, covering genotype combinations of NAT2*5/*5, NAT2*5/*6, NAT2*5/*7, NAT2*6/*6, NAT2*6/*7, and NAT2*7/*7. Acetylator status was inferred solely from the NAT2 genotype. Isoniazid concentrations, acetylisoniazid concentrations, and other pharmacokinetic measurements were not systematically captured in the analytic dataset and were not used to classify or validate acetylator status in the present study.

### Liver function data collection and outcome definition

Liver function tests at admission and during hospitalization were extracted from the medical records. Analyses of incident abnormalities were restricted to participants with normal liver function at admission. The study outcome was a new in-hospital liver function abnormality, defined as any of the following during hospitalization or at discharge: (1) alanine aminotransferase (ALT) or aspartate aminotransferase (AST) > 5 × the upper limit of normal (ULN); (2) alkaline phosphatase (ALP) > 2 × ULN; or (3) total bilirubin (TBIL) > 2 × ULN with any elevation in ALT or AST [21]. The available retrospective records did not permit consistent determination of the exact onset or duration of each abnormality, distinction between transient and persistent abnormalities, or formal attribution of causality to anti-tuberculosis treatment.

### Statistical analysis

All analyses were performed using R software (version 4.4.0). Categorical variables are presented as counts and percentages, and continuous variables as medians with interquartile ranges. Group comparisons used the chi-square test or Fisher’s exact test, as appropriate. Associations of participant characteristics with genotype-inferred NAT2 acetylator status were assessed using ordinal logistic regression and were interpreted as descriptive associations rather than determinants of inherited genotype.

For the liver-function outcome, binary logistic regression was used to estimate odds ratios (ORs) and 95% confidence intervals (CIs) in the primary analysis set (*n* = 554). Model 0 was unadjusted. Model 1, the primary adjusted model, included SA status, age per 10 years, sex, and recorded INH dose per 0.1 g. Model 2 additionally included pulmonary tuberculosis and INH susceptibility. The index day was defined as the date of the first recorded INH administration during the index hospitalization. Same-day exposure was defined as recorded rifampicin or pyrazinamide use on the index day. Post-index 24–48-hour exposure was defined as recorded rifampicin or pyrazinamide use during the period from 24 to 48 h after the first recorded INH administration; use recorded on the index day was not included in this variable. Secondary three-group models used SA as the reference: Model 3a included age per 10 years, sex, recorded INH dose per 0.1 g, and same-day rifampicin and pyrazinamide exposure; Model 3b included the same demographic and INH covariates together with rifampicin and pyrazinamide exposure during the post-index 24–48-hour window.

In the baseline-normal cohort (*n* = 690), factors associated with new in-hospital liver function abnormality were explored using univariable logistic regression; variables with *P* < 0.10 were entered into the multivariable model used in the source analysis. BMI and alcohol-use data were missing for more than half of participants, and exposure to hepatoprotective drugs was not systematically available. In addition, two records contained hepatitis B-related diagnostic text, but the available retrospective data did not permit reliable confirmation of active viral hepatitis. These variables were therefore not included in the primary adjusted model. A sensitivity analysis was performed after excluding the two records with hepatitis B-related diagnostic text. Among slow acetylators in the primary analysis set, each raw genotype was compared with all other slow-acetylator genotypes using a two-sided Fisher exact test without adjustment for multiple comparisons. Precision was summarized using event-rate 95% CIs, and the minimum detectable effect was approximated using the observed group sizes and non-SA event rate with a two-sample proportion test at two-sided α = 0.05 and 80% power. Sensitivity analyses included the original baseline-normal cohort and a diagnosis-text screen excluding two records with hepatitis B-related text. All tests were two-sided, and *P* < 0.05 was considered statistically significant; exploratory results were interpreted cautiously.

## Results

### Study population and baseline characteristics

**Fig. 1:**
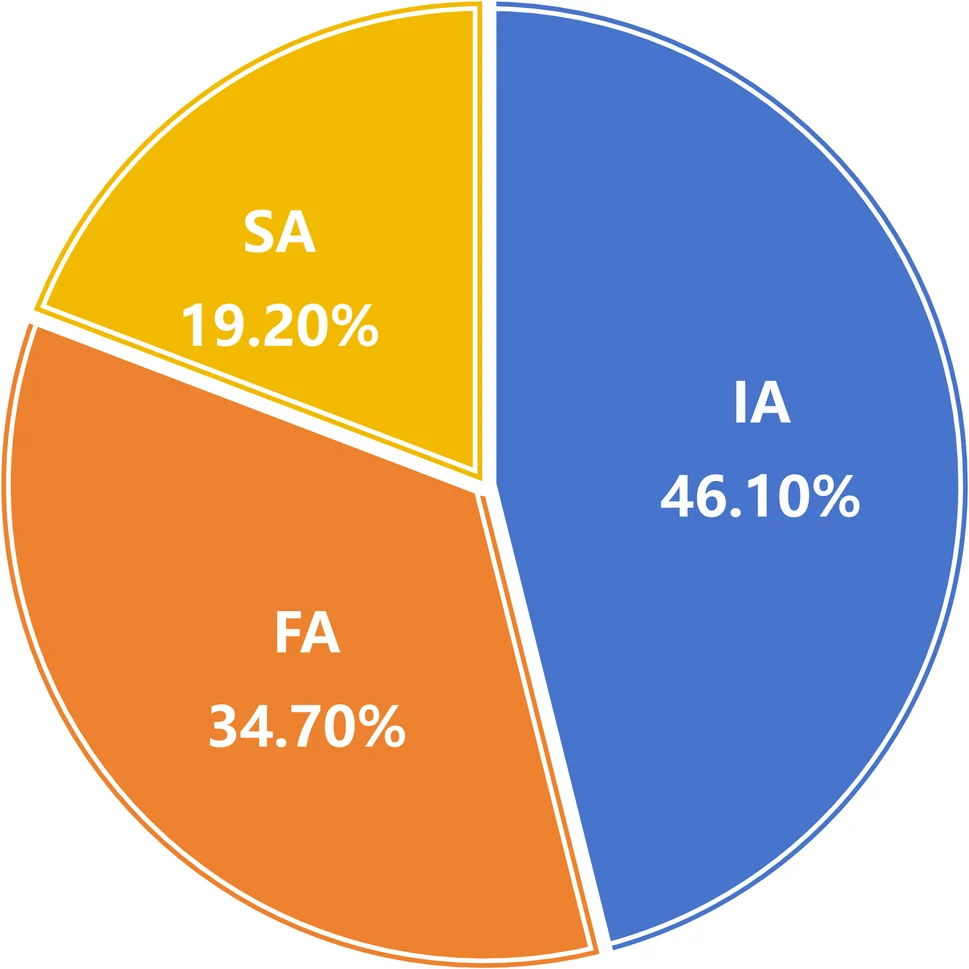
Distribution of NAT2 acetylator phenotypes in tuberculosis patients from a single center in Henan Province. FA, fast acetylator. IA, intermediate acetylator. SA, slow acetylator

Overall, 801 patients were included in the cohort (Table 1). The median age was 46.0 years (IQR, 29.0–64.0), 472 (58.9%) were male, and 657 (82.0%) had pulmonary tuberculosis. Genotype-inferred acetylator status was intermediate in 369 patients (46.1%), fast in 278 (34.7%), and slow in 154 (19.2%) (Fig. 1). Liver function at admission was normal in 690 patients (86.1%); 554 patients with normal baseline liver function and a recorded INH dose comprised the primary analysis set. BMI and alcohol-use data were missing for more than half of participants, and rifampicin and pyrazinamide exposure was unknown in 162 patients (20.2%).

**Table 1:** Patient general information

Characteristic	Total (*N* = 801)
Age, years, median (IQR)	46.00 (29.00, 64.00)
**Age**,** years**,** n (%)**	
< 20	65 (8.1)
20–39	276 (34.5)
40–59	217 (27.1)
≥ 60	243 (30.3)
**Sex**,** n (%)**	
Female	329 (41.1)
Male	472 (58.9)
**Region**,** n (%)**	
Zhengzhou	193 (24.1)
Xuchang	95 (11.9)
Zhoukou	81 (10.1)
Zhumadian	76 (9.5)
Shangqiu	52 (6.5)
Nanyang	41 (5.1)
Xinyang	40 (5.0)
Kaifeng	31 (3.9)
Pingdingshan	30 (3.7)
Jiaozuo	26 (3.2)
Luoyang	24 (3.0)
Xinxiang	23 (2.9)
Luohe	23 (2.9)
Anyang	21 (2.6)
Sanmenxia	17 (2.1)
Puyang	12 (1.5)
Jiyuan	11 (1.4)
Hebi	5 (0.6)
**Type of tuberculosis**,** n (%)**	
Pulmonary tuberculosis	657 (82.0)
Lymph node tuberculosis	46 (5.7)
Other tuberculosis	98 (12.2)
**INH susceptibility**,** n (%)**	
INH susceptible	113 (14.1)
INH resistant	25 (3.1)
INH susceptibility not tested	663 (82.8)
**Xpert**,** n (%)**	
Positive	351 (43.8)
Negative	254 (31.7)
Not tested	196 (24.5)
**QFT-Plus**,** n (%)**	
Positive	647 (80.8)
Negative	70 (8.7)
Not tested	84 (10.5)
**Mycobacterial culture**,** n (%)**	
Positive	301 (37.6)
Negative	318 (39.7)
Not tested	182 (22.7)
**Genotype-inferred acetylator status**,** n (%)**	
Slow acetylator	154 (19.2)
Intermediate acetylator	369 (46.1)
Fast acetylator	278 (34.7)
**Liver function at admission**,** n (%)**	
Normal	690 (86.1)
Abnormal	104 (13.0)
Not tested	7 (0.9)
**Smear**,** n (%)**	
Positive	160 (20.0)
Negative	414 (51.7)
Not tested	227 (28.3)
**INH dose**,** g**,** n (%)**	
Not used	155 (19.4)
0.1	9 (1.1)
0.2	8 (1.0)
0.3	591 (73.8)
0.4	22 (2.7)
0.5	11 (1.4)
0.6	5 (0.6)
Duration of INH use, days, median (IQR) [*n* = 646]	30.00 (10.25, 53.00)
**Duration of INH use**,** days**,** n (%)**	
≤ 30	327 (40.8)
> 30	319 (39.8)
Not used	155 (19.4)
Length of hospital stay, days, median (IQR)	37.00 (16.00, 60.00)
**Length of hospital stay**,** days**,** n (%)**	
≤ 30	332 (41.4)
> 30, ≤ 60	271 (33.8)
> 60	198 (24.7)
BMI, kg/m², median (IQR) [*n* = 340]	22.06 (19.82, 24.71)
**BMI**,** kg/m²**,** n (%)**	
Underweight	52 (6.5)
Normal	171 (21.3)
Overweight	96 (12.0)
Obesity	21 (2.6)
Missing	461 (57.6)
**Alcohol use**,** n (%)**	
No	328 (40.9)
Yes	16 (2.0)
Missing	457 (57.1)
**Rifampicin use on index day**,** n (%)**	
No	292 (36.5)
Yes	347 (43.3)
Unknown	162 (20.2)
**Pyrazinamide use during post-index hours 24–48**,** n (%)**	
No	560 (69.9)
Yes	79 (9.9)
Unknown	162 (20.2)
**Pyrazinamide use on index day**,** n (%)**	
No	325 (40.6)
Yes	314 (39.2)
Unknown	162 (20.2)
**Pyrazinamide use during post-index hours 24–48**,** n (%)**	
No	554 (69.2)
Yes	85 (10.6)
Unknown	162 (20.2)
**Hepatitis B-related diagnostic text**,** n (%)**	
No	799 (99.8)
Yes	2 (0.2)

Note: The index day was the date of the first recorded INH administration during the index hospitalization. The post-index 24–48-hour window covered the period from 24 to 48 h after the first recorded INH administration and did not include exposure recorded on the index day

### Distribution of NAT2 acetylator status

Baseline demographic, disease, and treatment characteristics did not differ significantly among the SA, IA, and FA groups (all *P* > 0.05; Table 2). Ordinal regression likewise showed no statistically significant associations between the evaluated participant characteristics and genotype-inferred acetylator status (Supplementary Table S1). BMI and alcohol use were interpreted descriptively because of substantial missingness.

**Table 2:** Baseline characteristics of different acetylation metabolism types

Characteristic	Genotype-inferred acetylator status			*P*
	Slow acetylator	Intermediate acetylator	Fast acetylator	
	(*n* = 154)	(*n* = 369)	(*n* = 278)	
Age, years, n (%)				0.196
< 20	10 (6.5)	27 (7.3)	28 (10.1)	
20–39	62 (40.3)	127 (34.4)	87 (31.3)	
40–59	46 (29.9)	101 (27.4)	70 (25.2)	
≥ 60	36 (23.4)	114 (30.9)	93 (33.5)	
Sex, n (%)				0.577
Male	85 (55.2)	221 (59.9)	166 (59.7)	
Female	69 (44.8)	148 (40.1)	112 (40.3)	
Region, n (%)				0.947
Zhengzhou	40 (26.0)	84 (22.8)	69 (24.8)	
Xuchang	19 (12.3)	42 (11.4)	34 (12.2)	
Zhoukou	19 (12.3)	35 (9.5)	27 (9.7)	
Zhumadian	16 (10.4)	35 (9.5)	25 (9.0)	
Shangqiu	7 (4.5)	25 (6.8)	20 (7.2)	
Other prefecture-level cities in Henan	53 (34.4)	148 (40.1)	103 (37.1)	
Type of tuberculosis, n (%)				0.496
Pulmonary tuberculosis	132 (85.7)	297 (80.5)	228 (82.0)	
Lymph node tuberculosis	9 (5.8)	20 (5.4)	17 (6.1)	
Other tuberculosis	13 (8.4)	52 (14.1)	33 (11.9)	
INH susceptibility, n (%)				0.521
INH susceptible	19 (12.3)	57 (15.4)	37 (13.3)	
INH resistant	7 (4.5)	8 (2.2)	10 (3.6)	
INH susceptibility not tested	128 (83.1)	304 (82.4)	231 (83.1)	
Liver function at admission, n (%)				0.553
Abnormal	24 (15.6)	44 (12.1)	36 (13.1)	
Normal	130 (84.4)	321 (87.9)	239 (86.9)	
INH dose, n (%)				0.729
≤ 0.3 g	33 (21.4)	64 (17.3)	58 (20.9)	
> 0.3 g	115 (74.7)	286 (77.5)	207 (74.5)	
Not used	6 (3.9)	19 (5.1)	13 (4.7)	
Duration of INH use, n (%)				0.348
≤ 30d	55 (35.7)	152 (41.2)	120 (43.2)	
> 30d	66 (42.9)	153 (41.5)	100 (36.0)	
Not used	33 (21.4)	64 (17.3)	58 (20.9)	
Length of hospital stay, n (%)				0.184
≤ 30d	55 (35.7)	149 (40.4)	128 (46.0)	
> 30d, ≤ 60d	52 (33.8)	130 (35.2)	89 (32.0)	
> 60d	47 (30.5)	90 (24.4)	61 (21.9)	
BMI, kg/m², n (%)				0.078
Underweight	12 (7.8)	21 (5.7)	19 (6.8)	
Normal	37 (24.0)	78 (21.1)	56 (20.1)	
Overweight	14 (9.1)	40 (10.8)	42 (15.1)	
Obesity	9 (5.8)	8 (2.2)	4 (1.4)	
Missing	82 (53.2)	222 (60.2)	157 (56.5)	
Alcohol use, n (%)				0.081
No	66 (42.9)	142 (38.5)	120 (43.2)	
Yes	7 (4.5)	5 (1.4)	4 (1.4)	
Missing	81 (52.6)	222 (60.2)	154 (55.4)	
Rifampicin use on index day, n (%)				0.752
No	54 (35.1)	137 (37.1)	101 (36.3)	
Yes	66 (42.9)	165 (44.7)	116 (41.7)	
Unknown	34 (22.1)	67 (18.2)	61 (21.9)	
Rifampicin use during post-index hours 24–48, n (%)				0.645
No	103 (66.9)	264 (71.5)	193 (69.4)	
Yes	17 (11.0)	38 (10.3)	24 (8.6)	
Unknown	34 (22.1)	67 (18.2)	61 (21.9)	
Pyrazinamide use on index day, n (%)				0.719
No	59 (38.3)	153 (41.5)	113 (40.6)	
Yes	61 (39.6)	149 (40.4)	104 (37.4)	
Unknown	34 (22.1)	67 (18.2)	61 (21.9)	
Rifampicin use during post-index hours 24–48, n (%)				0.279
No	110 (71.4)	257 (69.6)	187 (67.3)	
Yes	10 (6.5)	45 (12.2)	30 (10.8)	
Unknown	34 (22.1)	67 (18.2)	61 (21.9)	

Note: Data are n (%) unless otherwise indicated. P values compare SA, IA, and FA groups. BMI and alcohol-use data were missing for more than half of participants; viral hepatitis was rare. These variables are presented descriptively and were not considered fully controlled in the primary model. The index day was the date of the first recorded INH administration during the index hospitalization. The post-index 24–48-hour window did not include exposure recorded on the index day

### Association between NAT2 acetylator status and new in-hospital liver function abnormality

In the primary analysis set (*n* = 554), 143 patients developed a new in-hospital liver function abnormality. The event rate was 28.7% (29/101) among slow acetylators and 25.2% (114/453) among non-slow acetylators (crude OR, 1.20; 95% CI, 0.73–1.92; *P* = 0.462; Table 3). After adjustment for age, sex, and recorded INH dose, no statistically significant association was detected between slow acetylator status and the outcome (adjusted OR, 1.18; 95% CI, 0.72–1.89; *P* = 0.507). Further adjustment for pulmonary tuberculosis and INH susceptibility produced a similar estimate (OR, 1.18; 95% CI, 0.72–1.90; *P* = 0.505; Table 4).

**Table 3:** Event rates and crude associations

Comparison	NAT2 group	*N*	Events	Event rate	Crude OR (95%CI)	*P*
Primary	non-SA (IA + FA)	453	114	25.20%	reference	
Primary	SA	101	29	28.70%	1.20 (0.73, 1.92)	0.462
Secondary	SA	101	29	28.70%	reference	
Secondary	IA	265	70	26.40%	0.89 (0.54, 1.50)	0.658
Secondary	FA	188	44	23.40%	0.76 (0.44, 1.32)	0.323

**Table 4:** Logistic regression models

Model	Comparison	Adjustment variables	*N*	OR (95%CI)	*P*
Model 0: unadjusted	SA vs. non-SA	SA only	554	1.20 (0.73, 1.92)	0.462
Model 1: primary	SA vs. non-SA	SA + age/10 + sex + recorded INH dose/0.1 g	554	1.18 (0.72, 1.89)	0.507
Model 2: expanded sensitivity	SA vs. non-SA	Model 1 + pulmonary TB + INH susceptibility	554	1.18 (0.72, 1.90)	0.505
Model 3a: three-group adjusted — same-day exposure model	IA vs. SA	Age/10 + sex + recorded INH dose/0.1 g + same-day rifampicin + same-day pyrazinamide	554	0.90 (0.54, 1.52)	0.692
Model 3a: three-group adjusted — same-day exposure model	FA vs. SA	Age/10 + sex + recorded INH dose/0.1 g + same-day rifampicin + same-day pyrazinamide	554	0.76 (0.44, 1.33)	0.34
Model 3b: three-group adjusted —post-index 24–48-hour exposure model	IA vs. SA	Age/10 + sex + recorded INH dose/0.1 g + rifampicin within 48 h + pyrazinamide within 48 h	554	0.92 (0.56, 1.56)	0.763
Model 3b: three-group adjusted —post-index 24–48-hour exposure model	FA vs. SA	Age/10 + sex + recorded INH dose/0.1 g + rifampicin within 48 h + pyrazinamide within 48 h	554	0.78 (0.45, 1.37)	0.385

Note: Primary analysis set: participants with normal liver function at admission and a recorded INH dose (*n* = 554)

Model 0 was unadjusted. Model 1 adjusted for age per 10 years, sex, and recorded INH dose per 0.1 g. Model 2 additionally included pulmonary tuberculosis and INH susceptibility. Model 3a additionally adjusted for rifampicin and pyrazinamide exposure recorded on the index day; Model 3b additionally adjusted for rifampicin and pyrazinamide exposure during the post-index 24–48-hour window, excluding exposure recorded on the index day

Event rates were 26.4% in the IA group and 23.4% in the FA group, compared with 28.7% in the SA group. In the three-group models accounting for rifampicin and pyrazinamide exposure on the index day or during the post-index 24–48-hour window, adjusted ORs for IA and FA versus SA ranged from 0.76 to 0.92, and all 95% CIs included 1.0 (Table 4).

### Exploratory and sensitivity analyses

Among the 690 patients with normal liver function at admission, 177 developed the study outcome (Supplementary Table S2). In the multivariable analysis, the estimates for age 20–39 years (OR, 2.143; 95% CI, 0.996–4.614; *P* = 0.051) and INH use for > 30 days (OR, 1.427; 95% CI, 0.971–2.097; *P* = 0.070) did not reach statistical significance. IA and FA were also not significantly associated with the outcome when SA was the reference.

Within the SA group in the primary analysis set, 18 of 45 patients (40.0%) with the GA GA TT raw genotype pattern developed the outcome (Fisher *P* = 0.029); this exploratory comparison was not adjusted for multiple testing (Supplementary Table S3). Based on the observed sample sizes, the approximate minimum detectable effects at 80% power corresponded to ORs of 1.94 and 0.45 for risk-increasing and protective associations, respectively (Supplementary Table S4). Results were similar in the baseline-normal cohort (age- and sex-adjusted OR for SA versus non-SA, 1.30; 95% CI, 0.84–1.97; *P* = 0.225). The estimate was also materially unchanged after excluding the two records containing hepatitis B-related diagnostic text (adjusted OR, 1.29; 95% CI, 0.84–1.96; *P* = 0.231; Supplementary Table S5).

## Discussion

This study characterized the distribution of genotype-inferred NAT2 acetylator phenotypes and examined their association with new in-hospital liver function abnormality in patients with tuberculosis. In the overall cohort, intermediate acetylators were most common (46.1%), followed by fast (34.7%) and slow acetylators (19.2%). In the primary analysis set, the event rate was 28.7% in slow acetylators and 25.2% in non-slow acetylators. The adjusted estimate was close to the crude estimate (adjusted OR, 1.18; 95% CI, 0.72–1.89), and the results remained similar after additional adjustment and in sensitivity analyses. Thus, this study did not detect a statistically significant association between slow acetylator status and the study outcome; however, the confidence interval does not establish absence of an effect.

The acetylator distribution in this cohort is broadly consistent with the predominance of intermediate and fast acetylation reported in East Asian populations, whereas slow acetylation is more frequent in many African and European populations [15]. We observed no clear differences in acetylator distribution by sex or recorded region within the study population. These descriptive data may be useful for planning pharmacogenomic studies in central China, but they should not be interpreted as population estimates for Henan Province because all participants were recruited from a single specialist hospital and only genotyped patients were included.

The absence of a statistically significant association in our primary analysis contrasts with several previous studies and pooled analyses. In a Taiwanese cohort of 224 patients, slow acetylation was independently associated with antituberculosis drug-induced hepatitis (OR, 3.66; 95% CI, 1.58–8.49) [22]. Meta-analyses have also reported higher hepatotoxicity risk among slow acetylators, although pooled estimates and the quality of the contributing studies vary [19, 23, 24]. An important distinction is outcome definition. Our endpoint was any new liver function abnormality documented during hospitalization, rather than causally adjudicated anti-tuberculosis drug-induced liver injury (ATB-DILI). Contemporary guidance recommends defined biochemical thresholds, assessment of temporal change, exclusion of competing causes, and formal causality evaluation for ATB-DILI [21]. A broader and less specific endpoint may therefore dilute an association with genetically mediated isoniazid toxicity and partly explain differences from studies using stricter case definitions.

Statistical precision is also important when interpreting the primary result. The observed sample size provided approximately 80% power to detect an OR of 1.94 or greater for a risk-increasing association, whereas smaller or moderate effects could have remained undetected. Accordingly, the point estimate and its confidence interval are compatible with both little association and a clinically relevant increase in risk. Within the slow acetylator group, the GA GA TT raw genotype pattern had an event rate of 40.0% and a two-sided Fisher P value of 0.029. This finding should be considered hypothesis-generating because the comparison was unadjusted, involved small genotype-specific groups, and was not corrected for multiple testing. It should not outweigh the prespecified phenotype-level multivariable analyses.

Liver abnormalities during tuberculosis treatment are multifactorial. Adjustment for rifampicin and pyrazinamide exposure on the index day or during the post-index 24–48-hour window did not materially change the acetylator-status estimates, but residual confounding remains likely. BMI and alcohol-use data were missing for more than half of the cohort, viral hepatitis was rarely recorded, and systematic information on hepatoprotective agents and other potentially hepatotoxic medications was unavailable. We also lacked measurements of isoniazid exposure and variants in other relevant pathways, including CYP2E1 and glutathione S-transferases [18, 21, 25]. These limitations preclude attributing liver function abnormalities to NAT2 alone. In particular, because hepatoprotective treatment was not systematically captured, the present data cannot support the previous speculation that such treatment masked a true NAT2 association.

The exploratory baseline-normal analysis suggested higher odds of the outcome among patients aged 20–39 years and those receiving isoniazid for more than 30 days, but both associations were borderline after adjustment. These estimates are unstable because the reference group aged < 20 years was small. In addition, without a consistently recorded date of onset, duration of isoniazid treatment may partly reflect opportunity for inpatient detection rather than a causal exposure interval. Prospective studies with serial liver tests and time-to-event analysis are needed to distinguish treatment duration from monitoring and follow-up effects. Although NAT2-guided dosing has biological and pharmacokinetic plausibility [9, 10, 18], and a randomized Japanese trial reported fewer isoniazid-related liver injuries in slow acetylators and fewer early treatment failures in rapid acetylators with genotype-guided dosing [26], the clinical evidence remains limited [15]. Our observational study did not evaluate genotype-guided prescribing or systematically assess pharmacokinetic isoniazid exposure, microbiological response, treatment completion, relapse, or mortality and therefore cannot determine the clinical benefit of NAT2-guided dosing.

This study has strengths, including a relatively large genotyped cohort, restriction of the primary analysis to patients with normal baseline liver function and a recorded isoniazid dose, multivariable adjustment, and sensitivity analyses. This study has several limitations that should be considered when interpreting the findings. First, the retrospective single-center design may have introduced selection bias because only patients who underwent NAT2 genotyping were included. The clinical characteristics and outcomes of patients who did not undergo NAT2 testing could not be compared; therefore, the generalizability of the findings may be limited. In addition, despite multivariable adjustment and sensitivity analyses, residual confounding cannot be excluded because several potentially relevant factors, including body mass index, alcohol consumption, viral hepatitis status, rifampicin/pyrazinamide exposure, hepatoprotective medication use, and other hepatotoxic medications, were incompletely captured or unavailable.

Second, although the study included 801 patients, the statistical power was limited for detecting smaller or moderate associations. The minimum detectable effect analysis indicated approximately 80% power to identify a risk-increasing association with an OR of 1.94 or greater. Therefore, the absence of statistical significance should not be interpreted as evidence that NAT2 acetylator status has no clinically meaningful effect, as smaller effects cannot be excluded.

Third, the outcome evaluated in this study was new in-hospital liver function abnormality rather than causally adjudicated anti-tuberculosis drug-induced liver injury (ATB-DILI). Detailed information regarding temporal relationships between drug exposure and liver abnormalities, competing causes, treatment interruption, rechallenge, and formal causality assessment was not consistently available. Therefore, these findings should not be interpreted as direct evidence regarding the presence or absence of NAT2-related susceptibility to isoniazid-induced hepatotoxicity.

Fourth, NAT2 acetylator status was inferred from genotype and was not validated using pharmacokinetic measurements. Systematic measurements of isoniazid or acetylisoniazid concentrations were unavailable, and genotype-defined acetylator categories may not completely reflect individual drug exposure or metabolic phenotype. Prospective studies incorporating pharmacokinetic validation are required to clarify the clinical utility of NAT2-guided isoniazid dosing.

Finally, the slow-acetylator subgenotype analysis was exploratory and hypothesis-generating because of the small genotype-specific subgroups, lack of correction for multiple comparisons, and absence of independent validation. Moreover, longitudinal treatment outcomes, including microbiological response, treatment completion, treatment interruption or discontinuation, relapse, and mortality, were not consistently available. Future prospective multicenter studies with larger cohorts, standardized ATB-DILI assessment, pharmacokinetic evaluation, and longitudinal follow-up are warranted to further clarify whether NAT2 variation contributes to isoniazid-associated liver toxicity and whether genotype-guided dosing strategies may have clinical utility.

## Conclusions

In this single-center retrospective cohort, intermediate acetylators were the most common genotype-inferred NAT2 phenotype, followed by fast and slow acetylators. Slow acetylator status was not significantly associated with new in-hospital liver function abnormality after multivariable adjustment, although smaller or moderate effects could not be excluded. Because the outcome was not causally adjudicated ATB-DILI and systematic pharmacokinetic and longitudinal treatment data were unavailable, these findings neither establish nor refute the clinical benefit of NAT2-guided dosing. Prospective multicenter studies using standardized ATB-DILI definitions, scheduled liver monitoring, pharmacokinetic measurements, and longitudinal efficacy and safety outcomes are needed before routine implementation can be considered.

## Supplementary Information

Below is the link to the electronic supplementary material.

Supplementary Material 1

## Data Availability

The datasets used and/or analysed during the current study are available from the corresponding author on reasonable request.

## Abbreviations

FA: Fast acetylator
IA: Intermediate acetylator
INH: Isoniazid
IQR: Interquartile range
NAT2: N-acetyltransferase 2
SA: Slow acetylator
SNPs: Single nucleotide polymorphisms
TB: Tuberculosis
WHO: World Health Organization
