# Supplementary material for: The distribution of N-acetyltransferase 2 genotypes in a cohort of tuberculosis patients in a single center in Henan Province of China
Source: BMC Infect Dis. 2026 Sep 22;26:1988. doi: 10.1186/s12879-026-14424-x (PMC13761129; doi:10.1186/s12879-026-14424-x)
Supplement: Supplementary file 1 [file 12879_2026_14424_MOESM1_ESM.docx]

**Supplementary materials**

**Table S1. Associations of participant characteristics with genotype-inferred NAT2 acetylator status**

| **Variable** | **Univariate analysis  Odds ratio (95% CI)** | **P value** | **Multivariate analysis Odds ratio (95% CI)** | **P value** |
| --- | --- | --- | --- | --- |
| **Age** |  |  |  |  |
| <20 | 1 |  |  |  |
| 20-39 | 0.607 (0.363, 1.014) | 0.056 |  |  |
| 40-59 | 0.639 (0.377, 1.080) | 0.094 |  |  |
| ≥60 | 0.876 (0.522, 1.471) | 0.616 |  |  |
| **Sex** |  |  |  |  |
| Female | 1 |  |  |  |
| Male | 1.106 (0.849, 1.440) | 0.457 |  |  |
| **Region** |  |  |  |  |
| Zhengzhou | 1 |  |  |  |
| Xuchang | 1.018 (0.640, 1.619) | 0.939 |  |  |
| Zhoukou | 0.874 (0.534, 1.429) | 0.591 |  |  |
| Zhumadian | 0.915 (0.575, 1.455) | 0.727 |  |  |
| Shangqiu | 1.269 (0.776, 2.076) | 0.413 |  |  |
| Other cities/counties in Henan | 1.022 (0.643, 1.626) | 0.898 |  |  |
| **Type of tuberculosis** |  |  |  |  |
| Pulmonary tuberculosis | 1 |  |  |  |
| Lymph node tuberculosis | 1.080 (0.614, 1.900) | 0.789 |  |  |
| Other tuberculosis | 1.130 (0.765, 1.669) | 0.359 |  |  |
| **INH susceptibility** |  |  |  |  |
| INH susceptible | 1 |  |  |  |
| INH resistant | 0.945 (0.406, 2.201) | 0.896 |  |  |
| INH susceptibility not tested | 1.002 (0.693, 1.450) | 0.990 |  |  |
| **Liver function at admission** |  |  |  |  |
| Normal | 1 |  |  |  |
| Abnormal | 1.106 (0.748, 1.635) | 0.615 |  |  |

Note: Odds ratios were estimated using ordinal multinomial Logistic regression. Results describe associations and should not be interpreted as participant characteristics affecting inherited genotype.

**Table S2. Factors associated with in-hospital liver function abnormality in the baseline-normal cohort**

| **Variable** | **Univariate analysis  Odds ratio (95% CI)** | **P value** | **Multivariate analysis Odds ratio (95% CI)** | **P value** |
| --- | --- | --- | --- | --- |
| **Age** |  |  |  |  |
| <20 | 1 |  | 1 |  |
| 20-39 | 2.183 (1.017, 4.689) | 0.045 | 2.143 (0.996, 4.614) | 0.051 |
| 40-59 | 2.036 (0.930, 4.454) | 0.075 | 2.001 (0.912, 4.391) | 0.084 |
| ≥60 | 1.571 (0.719, 3.431) | 0.257 | 1.557 (0.712, 3.407) | 0.268 |
| **Sex** |  |  |  |  |
| Female | 1 |  |  |  |
| Male | 0.894 (0.633, 1.262) | 0.524 |  |  |
| **Type of tuberculosis** |  |  |  |  |
| Pulmonary tuberculosis | 1 |  |  |  |
| Lymph node tuberculosis | 0.480 (0.198, 1.164) | 0.104 |  |  |
| Other | 1.255 (0.769, 2.049) | 0.364 |  |  |
| **Genotype-inferred acetylator status** |  |  |  |  |
| Slow acetylator | 1 |  |  |  |
| Intermediate acetylator | 0.801 (0.510, 1.257) | 0.334 |  |  |
| Fast acetylator | 0.714 (0.442, 1.154) | 0.169 |  |  |
| **INH dose** |  |  |  |  |
| ≤0.3g | 1 |  |  |  |
| >0.3g | 1.059 (0.686, 1.635) | 0.795 |  |  |
| Not used | 0.783 (0.294, 2.083) | 0.624 |  |  |
| **Duration of INH use** |  |  |  |  |
| ≤30d | 1 |  | 1 |  |
| >30d | 1.461 (0.996, 2.144) | 0.052 | 1.427 (0.971, 2.097) | 0.070 |
| Not used | 1.167 (0.722, 1.885) | 0.529 | 1.108 (0.683, 1.797) | 0.679 |

Note: Secondary/exploratory analysis of 690 participants with normal liver function at admission; 177 developed the study outcome.

Variables with P<0.10 in univariable analysis were entered into the multivariable model in the source report.

**Table S3. Exploratory analysis of raw genotypes among slow acetylators**

| **Raw genotype** | **N** | **Events** | **Event rate** | **Fisher P** |
| --- | --- | --- | --- | --- |
| GA GA TT | 45 | 18 | 40.0% | 0.029 |
| AA GG TT | 22 | 3 | 13.6% | 0.110 |
| GG AA TT | 15 | 4 | 26.7% | 1.000 |
| GA GG TC | 10 | 2 | 20.0% | 0.720 |
| GG GA TC | 9 | 2 | 22.2% | 1.000 |

Note: Restricted to slow acetylators in the primary analysis set (n=101). Each two-sided Fisher exact test compared one raw genotype with all other slow-acetylator genotypes.

No adjustment was made for multiple comparisons; results are exploratory.

**Table S4. Precision and minimum detectable effect**

| **Group/parameter** | **N** | **Events/assumption** | **Event rate (95%CI)** | **Result** |
| --- | --- | --- | --- | --- |
| SA | 101 | 29 | 28.7% [20.4%, 38.7%] |  |
| non-SA | 453 | 114 | 25.2% [21.3%, 29.5%] |  |
| 80% power MDE |  | α=0.05; power=80% | Detectable SA rate: 39.5% / 13.2% | Risk-increasing: RD=+14.3%, OR=1.94; protective: RD=−12.0%, OR=0.45 |

Note: The MDE was approximated from the observed primary-analysis sample sizes and non-SA event rate using a two-sample proportion test with two-sided α=0.05 and 80% power.

This analysis describes detectable effect size and does not establish equivalence or absence of association.

**Table S5. Exclusion of hepatitis B-related diagnostic-text records**

| **Analysis** | **Comparison/model** | **OR (95%CI)** | **P** |
| --- | --- | --- | --- |
| Original baseline-normal cohort | SA vs non-SA; age/sex adjusted | 1.30 (0.84, 1.97) | 0.225 |
| Diagnosis text screen | SA vs non-SA; Model 1 covariates | 1.29 (0.84, 1.96) | 0.231 |

Note: The original baseline-normal cohort included all patients with normal baseline liver function, including patients recorded as not receiving INH. The sensitivity analysis was restricted to the primary analysis cohort after excluding two records containing hepatitis B-related diagnostic text (n=552). The available retrospective data did not permit reliable confirmation of active viral hepatitis from diagnosis text alone.
